# Effects of phycocyanin in modulating the intestinal microbiota of mice

**DOI:** 10.1002/mbo3.825

**Published:** 2019-03-25

**Authors:** Yuanyuan Xie, Wenjun Li, Limeng Zhu, Shixiang Zhai, Song Qin, Zhenning Du

**Affiliations:** ^1^ Yantai University Yantai China; ^2^ Yantai Institute of Coastal Zone Research, Chinese Academy of Sciences Yantai China; ^3^ Present address: Yantai University Yantai China; ^4^ Present address: Yantai Institute of Coastal Zone Research, Chinese Academy of Sciences Yantai China; ^5^ Present address: Institute of Process Engineering, Chinese Academy of Sciences Beijing China; ^6^ Present address: Yantai Institute of Coastal Zone Research, Chinese Academy of Sciences Yantai China

**Keywords:** 16S rRNA high‐throughput sequencing, intestinal barrier, intestinal microbiota, phycocyanin

## Abstract

The health‐promoting effects of phycocyanin (PC) have become widely accepted over the last two decades. In this study, we investigated the effects of different doses of PC in modulating the intestinal microbiota and the intestinal barrier in mice. Six‐week‐old male C57BL/6 mice were treated with PC for 28 days. Fecal samples were collected before and after PC intervention, and the microbiota were analyzed by 16S rRNA high‐throughput sequencing. Bacterial abundance and diversity increased after PC intervention. Saccharolytic bacteria of the families Lachnospiraceae and Ruminococcaceae, which can produce butyric acid, increased after PC treatment. The family Rikenellaceae, which contains hydrogen‐producing bacteria, also increased after PC intervention. The PC treatment reduced intestinal permeability and increased the intestinal barrier function, as demonstrated by hematoxylin–eosin staining and reduced serum lipopolysaccharide levels. The modulating effects on the intestinal microbiota were more favorable in the low‐dose PC group.

## 1 INTRODUCTION

The mammalian intestinal microbiota is highly complex and in dynamic equilibrium (Friche & Prado, 2017), and the balance of the intestinal microbiota is crucial to maintaining the host's health. With the development of 16S rRNA high‐throughput sequence technology (Rooks & Garrett, 2016), numerous chronic diseases, such as obesity (Renson & Manfroid, 2013), diabetes (Tilg & Moschen, 2014), cardiovascular disease (Zhu et al., 2016), and chronic kidney disease (Vaziri, Zhao, & Pahl, 2016), have been shown to be related to the gut environment. Diet is a key factor influencing the intestinal environment (Power, O'Toole, Stanton, Ross, & Fitzgerald, 2014). The intake of the three major dietary nutrients (carbohydrates, proteins, and fats) can significantly affect the composition of the microbiota (Scott, Gratz, Sheridan, Flint, & Duncan, 2013).

Phycocyanin (PC) is a light‐harvesting protein in the genus *Arthrospiraplatensis*, which participates in algal photosynthesis. It is an excellent natural dietary pigment (Li et al., 2017). The role of PC in health promotion has become widely accepted over the past two decades (Wu et al., 2016). Phycocyanin reportedly has many biological functions, including as an antitumor, anti‐inflammatory, and immunity‐enhancing agent (Jiang et al., 2017). Undigested proteins can be fermented by the intestinal microbiota. According to previous studies, proteins promote the growth of intestinal bacteria, and many of the nutrients available to these bacteria in the intestine derive from undigested proteins from the host's diet (Consortium et al., 2012).

The moderate restriction of dietary protein altered the composition of the gut microbiota and improved the ileal barrier function of adult pigs (Chen et al., 2018). The microbial composition and a wide range of microbial metabolites played a complex role in various host processes, such as energy harvesting, recovery from inflammation and infection, resistance to autoimmunity, and endocrine signaling, which affect brain function through the intestinal–brain axis (Han et al., 2016; Hollister, Gao, & Versalovic, 2014). The small intestinal and colonic microbes are also considered potential sources of amino acids in animals (Miller & Ullrey, 1987).

Mouse and human genes have a high degree of similarity (99% of genes in mice have homologues in the human genome). In this study, male C57BL/6 mice were selected as an experimental model and 16S rRNA high‐throughput sequencing was used to analyze the effects of PC treatment on the intestinal microbiota.

## 2 MATERIALS AND METHODS

### 2.1 Materials

Phycocyanin was purchased from King Dnarmse Spirulina Company (Fuqing, China). Maintenance diets for the mice were purchased from Pengyue Laboratory Animal Company (Jinan, China). Table 1 shows the daily diet of the mice.

**Table 1:** Nutrient content of the basal diet

Ingredient	Percentage (%)
Water	≤8.0
Crude protein	≥18.0
Crude fat	≥4.0
Crude fiber	≤5.0
Coarse ash	≤6.5
Calcium	1.2–1.4
Phosphorus	0.8–1.0

### 2.2 Animals and sample collection

A total of 27 male, 6‐week‐old, specific‐pathogen‐free C57BL/6 mice were used in this study. The study design was approved by the Animal Care and Maintenance Committee of Shandong International Biotechnology Park (SCXK 20140007), and adhered to the guidelines of the Institute of Health/Institutional Animal Care and Use Committee of Binzhou Medical University. All animals were allowed 1 week to acclimatize to 23 ± 2°C and 50%–60% humidity, with free access to food.

After acclimatization for 1 week, the mice were randomly separated into three groups: control group (*n* = 9), 50 mg/kg PC group (*n* = 9), and 100 mg/kg PC group (*n* = 9), and each group of mice was housed in a single cage. The 50 mg/kg PC group was given 5 mg L^−1^ day^−1^ PC by gavage; the 100 mg/kg PC group was given 10 mg L^−1^ day^−1^ PC by gavage; and the control group was given an equivalent volume of deionized water by gavage, once a day. Mouse bodyweights were measured at the beginning (0 weeks) and end (4 weeks) of the experiment. At the start and end of the experimental period (28 days), the mice were transferred individually to separate sterilized cages and their feces were collected. The samples were immediately stored at −80°C for the subsequent sequencing of the V3 and V4 regions of the bacterial 16S rRNA. At the end of the experiment, blood samples were collected, allowed to stand at 4°C for 30 min, and then centrifuged at 5,000*g* for 10 min at 4°C. The supernatants were collected and stored at −80°C. At the end of the experiment, ileal and colonic samples were collected from the mice and stored at −80°C until analysis.

### 2.3 Extraction of genomic DNA

The fecal samples were thawed on ice and the total genomic DNA was extracted with the QIAamp DNA Stool Mini Kit (Qiagen, Germany), according to the manufacturer's instructions. Agarose gel electrophoresis was used to determine the purity and concentration of the DNA. An appropriate amount of sample was diluted to 1 ng/μl with sterile water.

### 2.4 Amplicon generations

The bacterial universal V3–V4 region of the 16S rRNA gene was amplified. Genomic DNA was used as the template for PCR, with specific primers (338F and 806R) containing a barcode, Phusion® High‐Fidelity PCR Master Mix with GC buffer (New England Biolabs), and high‐efficiency fibrillation enzyme. The thermal cycling conditions were 95°C for 2 min; 30 cycles of 95°C for 30 s, 55°C for 30 s, and 72°C for 30 s; and a final extension at 72°C for 5 min.

The PCR products were detected with 2% agarose gel electrophoresis and purified with the AxyPrep DNA Purification Kit (Axygen Biosciences, Union City, CA, USA). The PCR products were then visualized on agarose gels and quantified with PicoGreen dsDNA Quantitation Reagent (Invitrogen, Carlsbad, CA, USA) and a QuantiFluor‐ST Fluorometer (Promega, USA).

### 2.5 Illumina sequencing

Purified amplicons were pooled and analyzed with the Illumina MiSeq platform (Majorbio, Shanghai, China), according to standard protocols for equimolar and paired‐end sequencing (2 × 300).

### 2.6 Data analysis

Fragments of paired‐ended sequencing were spliced with FLASH, and the clustering of operational taxonomic units (OTUs) and species classification were performed with a 97% similarity threshold. The RDP classifier was used to annotate the representative sequences of each OTU to obtain the corresponding species information and species‐based abundance distribution. The sequences were analyzed with the QIIME software, and α‐ and β‐diversities were analyzed with the Perl and R software to determine the species richness and the evenness of the samples. The samples were weighted with principal coordinates analysis (PCoA) based on the UniFrac distances, and were clustered to identify the differences in the community structures of the different samples and groups.

### 2.7 Intestinal morphology

The intestinal morphology was analyzed with hematoxylin–eosin (HE) staining. Ileal and colonic tissue samples were immediately fixed in polyoxymethylene after collection, for intestinal morphometry. The tissues were dehydrated and embedded according to standard procedures. The tissues in the paraffin blocks were cut into 4‐µm sections, stained with HE, and examined with a conventional BX‐51M microscope (Olympus). Image‐Pro Plus 5 software was used to observe the intestinal and colonic intestinal morphology.

### 2.8 Estimation of serum lipopolysaccharide (LPS) levels

The levels of serum LPS were determined with an enzyme‐linked immunosorbent assay (ELISA; Shanghai ELISA Kit). The procedure was performed according to the manufacturer's guidelines. The optical density of each well was determined at 450 nm with a M200 automatic enzyme label analyzer (Tecan, USA).

### 2.9 Statistical analysis

A homogeneity test of variance was performed before the difference analysis. All data are shown as means ± *SD*s. The differences between the different groups were tested by one‐way ANOVA (Tukey's test). All statistical analyses were performed with the SPSS 19.0 software, and *p* < 0.05 was considered to indicate significant differences.

## 3 RESULTS

### 3.1 Mouse bodyweights

**Figure 1:**
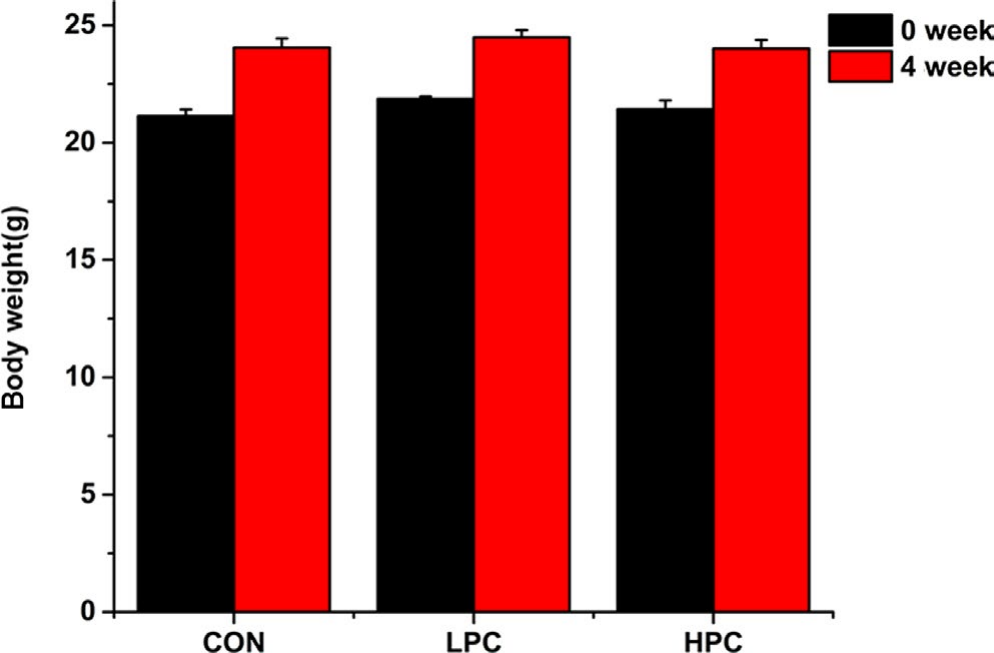
Bodyweights of the mice in the different groups after intervention for 4 weeks (*n* = 6 per group)

The average bodyweights of the three groups of mice increased in the 4‐week experimental period, but there was no significant difference between the control and experimental groups (Figure 1).

### 3.2 Sequencing data and α‐diversity

**Figure 2:**
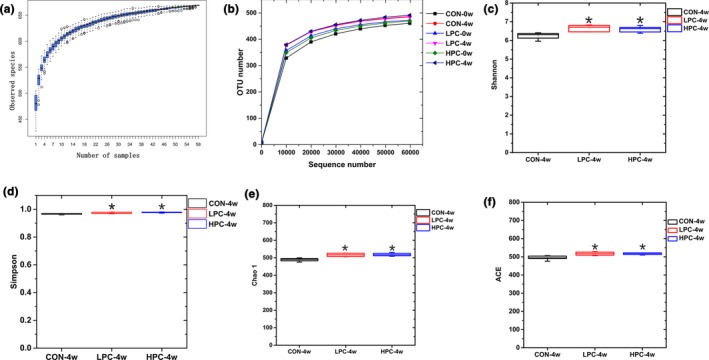
α‐Diversity and similarity of the intestinal microbiota communities in mice after PC interventions for 4 weeks. (a) Species accumulation boxplot for each sample. (b) Rarefaction curve for each sample. (c) Bacterial diversity in the gut estimated with the Shannon index. (d) Bacterial diversity in the gut estimated with the Simpson's index. (e) Bacterial richness in the gut estimated with the Chao1 value. (f) Bacterial richness in the gut estimated with the ACE value. CON‐0w, blank control group, week 0; CON‐4w, blank control group, week 4; LPC‐0w, 50 mg/kg PC group, week 0; LPC‐4w, 50 mg/kg PC group, week 4; HPC‐0w, 100 mg/kg PC group, week 0; HPC‐4w, 100 mg/kg PC group, week 4 (*n* = 6 per group). All data are shown as means ± *SD*s. Data were analyzed with one‐way ANOVA (Tukey's test), *indicates a significant difference compared with the control group (*p* < 0.05)

After size filtering, quality control, and chimera removal, 4,125,943 valid sequences were obtained. A species accumulation boxplot and sample rarefaction curve describe the increase in species diversity as the sample size increased. The species accumulation boxplot and sample rarefaction curve both plateaued, indicating that the experimental sequencing data were sufficient to reflect the microbial information in the samples (Figure 2a,b). We determined the bacterial richness and diversity of the intestinal microbiota in the mice before and after PC treatment. The Shannon and Simpson indices were higher in the PC groups after the intervention than in the control group (Figure 2c,d). The species richness was also higher in the PC intervention groups than in the control group, as shown by the Chao1 and ACE indices (Figure 2e,f).

### 3.3 Changes in intestinal microbial communities after PC treatment

**Figure 3:**
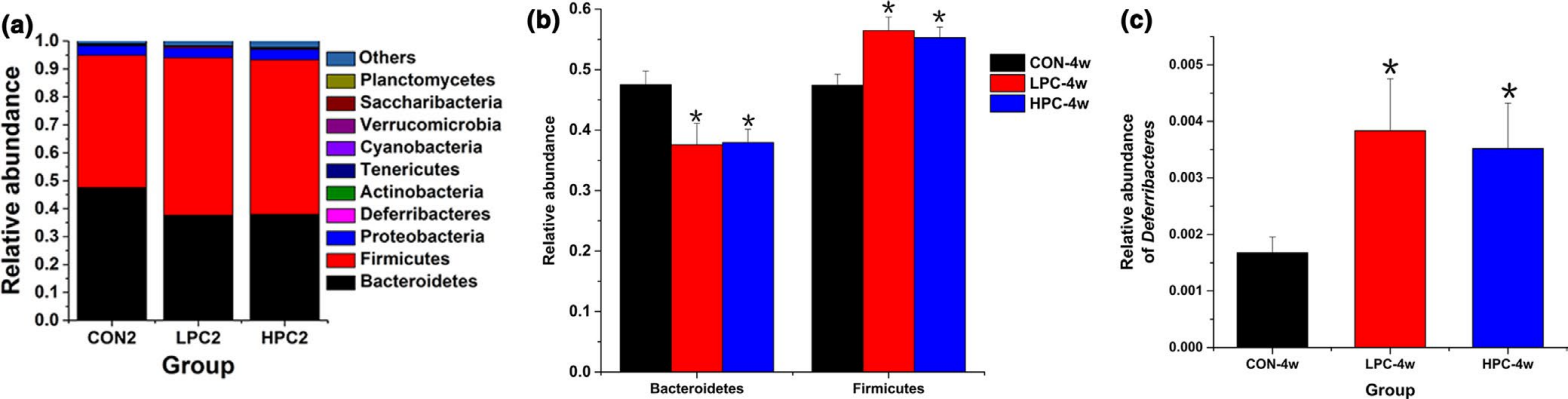
Effects of PC on the intestinal bacterial community structure at the phylum level. (a) Major intestinal microbiota distributions at the phylum level after PC intervention. (b) Relative abundances of the phyla Bacteroidetes and Firmicutes in the intestinal microbiota of the different groups. (c) Relative abundances of the phylum Deferribacteres in the intestinal microbiota of the different groups. CON‐4w, blank control group, week 4; LPC‐4w, 50 mg/kg PC group, week 4; HPC‐4w, 100 mg/kg PC group, week 4 (*n* = 6 per group). All data are shown as means ± *SD*s. Data were analyzed by one‐way ANOVA (Tukey's test); *indicates a significant difference compared with the control group (*p* < 0.05)

Firmicutes, Bacteroidetes, Proteobacteria, Deferribacteres, Actinobacteria, and Tenericutes were the most abundant phyla, and Firmicutes and Bacteroidetes were the dominant bacteria, accounting for more than 90% of all bacteria (Figure 3a). The relative abundance of Bacteroidetes decreased significantly (*p* < 0.05) after PC interventions for 4 weeks, whereas the abundance of Firmicutes increased significantly (*p* < 0.05) (Figure 3a,b). The relative abundance of Deferribacteres increased significantly (*p* < 0.05) after PC interventions for 4 weeks (Figure 3c), and the low‐dose PC group showed a greater increase than the high‐dose group. The other bacterial phyla remained relatively constant during the experiment (Figure 3a).

**Figure 4:**
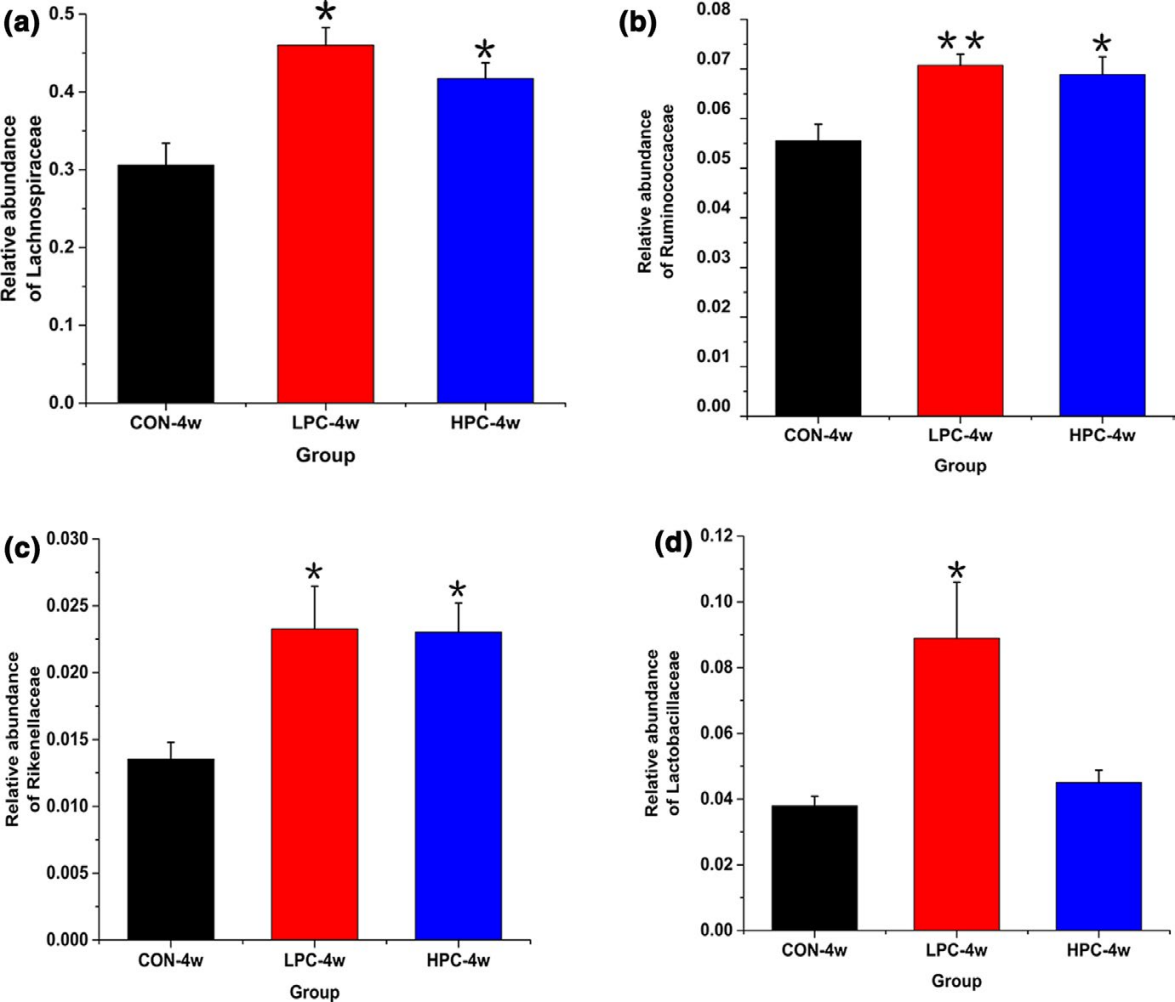
Mean changes in the relative abundances of bacterial families in fecal samples after PC interventions. (a) Family Lachnospiraceae, (b) family Ruminococcaceae; (c) family Rikenellaceae; (d) family Lactobacillaceae. CON‐4w, blank control group, week 4; LPC‐4w, 50 mg/kg PC group, week 4; HPC‐4w, 100 mg/kg PC group, week 4 (*n* = 6 per group). All data are shown as means ± *SD*s. Data were analyzed with one‐way ANOVA (Tukey's test). *indicates a significant difference compared with the control group (*p* < 0.05); **indicates an extremely significant difference compared with the control group (*p* < 0.01)

At the family level, the relative abundance of Lachnospiraceae, Ruminococcaceae, Rikenellaceae, and Lactobacillaceae was significantly altered after PC intervention (Figure 4). The abundance of Lachnospiraceae (phylum Firmicutes) was highest and increased significantly (*p* < 0.05) after PC interventions for 4 weeks (Figure 4a), and low‐dose PC had a more significant effect. The abundance of the families Ruminococcaceae and Rikenellaceae also increased significantly (*p* < 0.05) in the PC‐treated groups compared with the control group (Figure 4b,c), and the low‐dose PC group showed a greater effect than the high‐dose PC group. The family Lactobacillaceae increased dramatically in the low‐dose PC group (*p* < 0.05), whereas no significant change was observed in the high‐dose PC group (Figure 4d). Other bacterial families remained relatively constant during the experiment.

**Figure 5:**
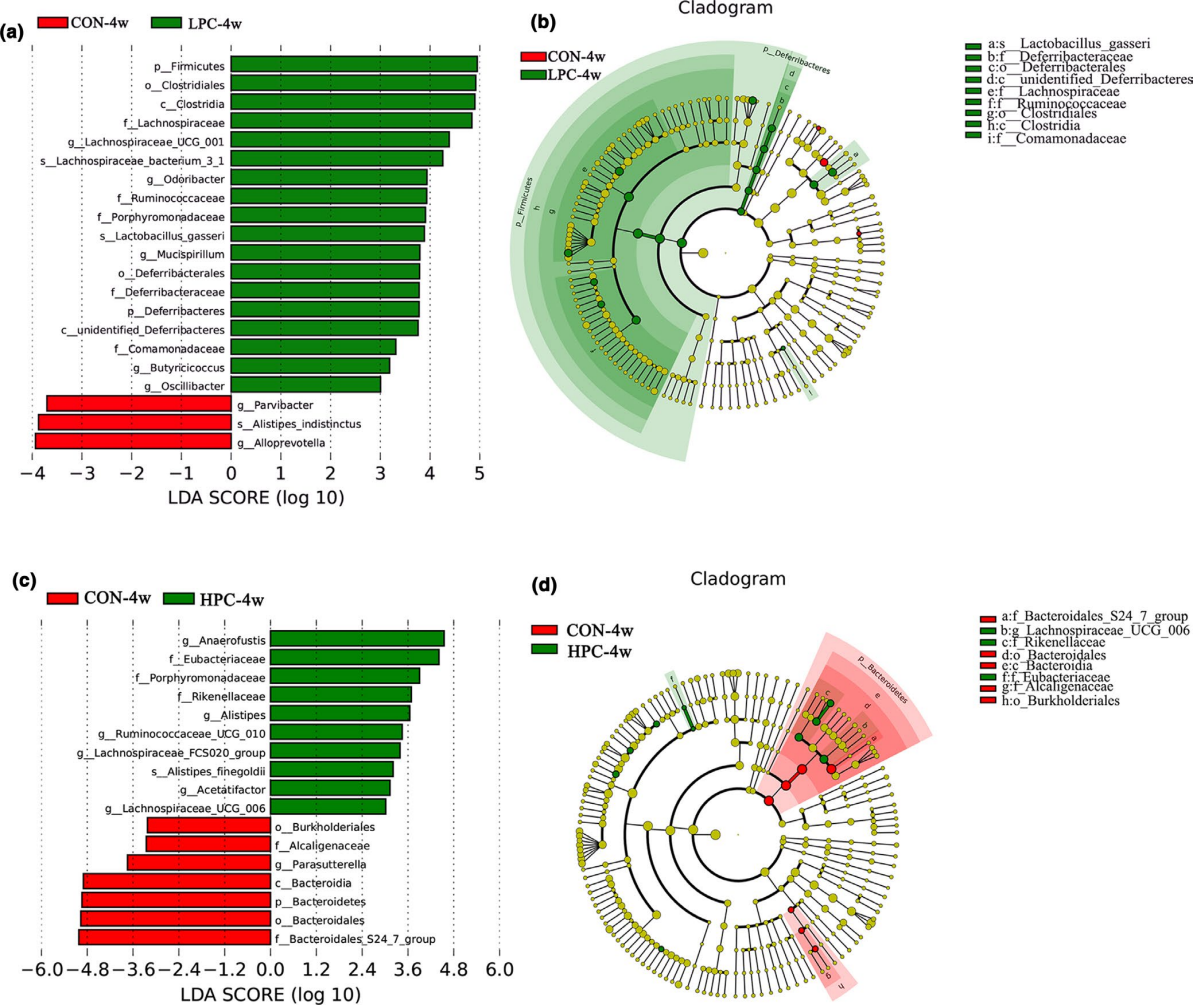
Key phylotypes in response to different doses of PC. (a) LEfSe comparison of the gut microbiota between CON‐4w and LPC‐4w, with a LDA score of greater than ±3.0. The length of the bar represents the LDA score. (b). Cladogram generated from the LEfSe analysis. (c). LEfSe comparison of gut microbiota in CON‐4w and HPC‐4w, with an LDA score of greater than ±3.0. The length of the bar represents the LDA score. (d) Cladogram generated from the LEfSe analysis. CON‐4w, blank control group, week 4; LPC‐4w, 50 mg/kg PC group, week 4; HPC‐4w, 100 mg/kg PC group, week 4 (*n* = 5 per group)

To identify biomarker bacteria in each group, we used linear discriminant analysis effect size (LEfSe) and set the linear discriminant analysis (LDA) score to greater than ±3 (Figure 5). The LPC‐4w group had 18 unique OTUs, and the CON‐4w group had three unique OTUs. Lactobacillaceae, Lachnospiraceae, and Ruminococcaceae were prevalent in the low‐dose PC group. The HPC‐4w group had 10 unique OTUs, and the CON‐4w group had seven unique OTUs*. *Lachnospiraceae and Rikenellaceae were prevalent in the high‐dose PC group. The intestinal microbiota increased more significantly in the low‐dose group than in the high‐dose group.

**Figure 6:**
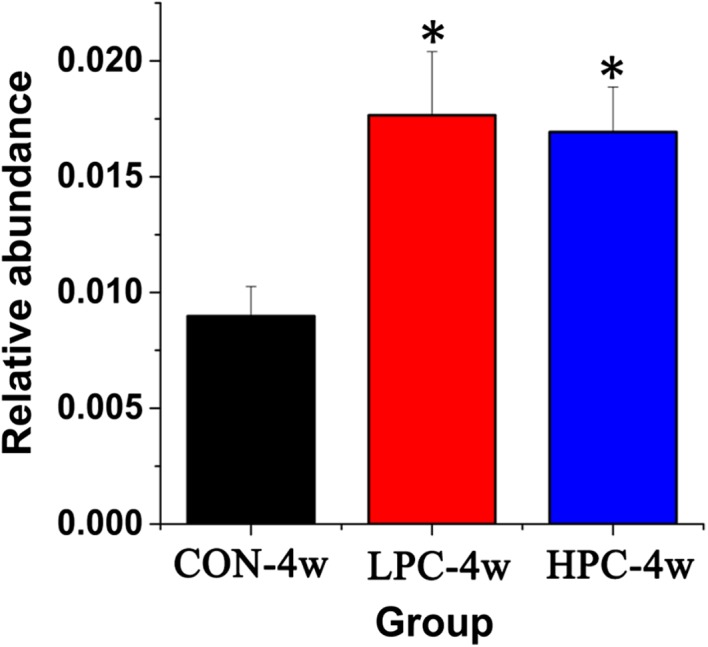
Mean changes in the relative abundance of the genus *Alistipes* in fecal samples after PC intervention. CON‐4w, blank control group, week 4; LPC‐4w, 50 mg/kg PC group, week 4; HPC‐4w, 100 mg/kg PC group, week 4 (*n* = 6 per group). All data are shown as means ± *SD*s. Data were analyzed by one‐way ANOVA (Tukey's test); *indicates a significant difference compared with the control group (*p* < 0.05)

At the genus level, the relative abundance of *Alistipes* (phylum Bacteroidetes, family Rikenellaceae) increased significantly (*p* < 0.05) after PC interventions for 4 weeks (Figure 6).

### 3.4 PCoA after administration of PC

**Figure 7:**
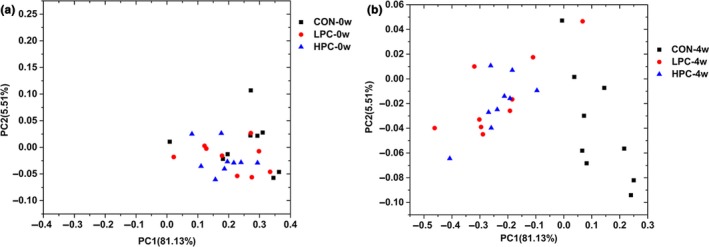
Separation of intestinal microbiota based on PC interventions. Principal coordinates analysis (PCoA) of the control group and PC groups at time point 0 and week 4 based on weighted UniFrac distances. (a) PCoA analysis of different groups at the beginning of the experiment. (b) PCoA analysis of the different groups at the end of the experiment. CON‐0w, blank control group, week 0; CON‐4w, blank control group, week 4; LPC‐0w, 50 mg/kg PC group, week 0; LPC‐4w, 50 mg/kg PC group, week 4; HPC‐0w, 100 mg/kg PC group, week 0; HPC‐4w, 100 mg/kg PC group, week 4 (*n* = 9 per group)

To explain the effects of PC on the variations in the intestinal microbiota, we conducted PCoA. A PCoA plot was generated based on the bacterial genera (Figure 7). The percentage of variation (81.13%) indicated that the separation of the fecal microbiota between the groups was caused by PC intervention. The composition of the fecal microbiota in each group was similar before PC intervention, and the differences between the groups occurred after PC treatment for 4 weeks.

### 3.5 Intestinal morphology

**Figure 8:**
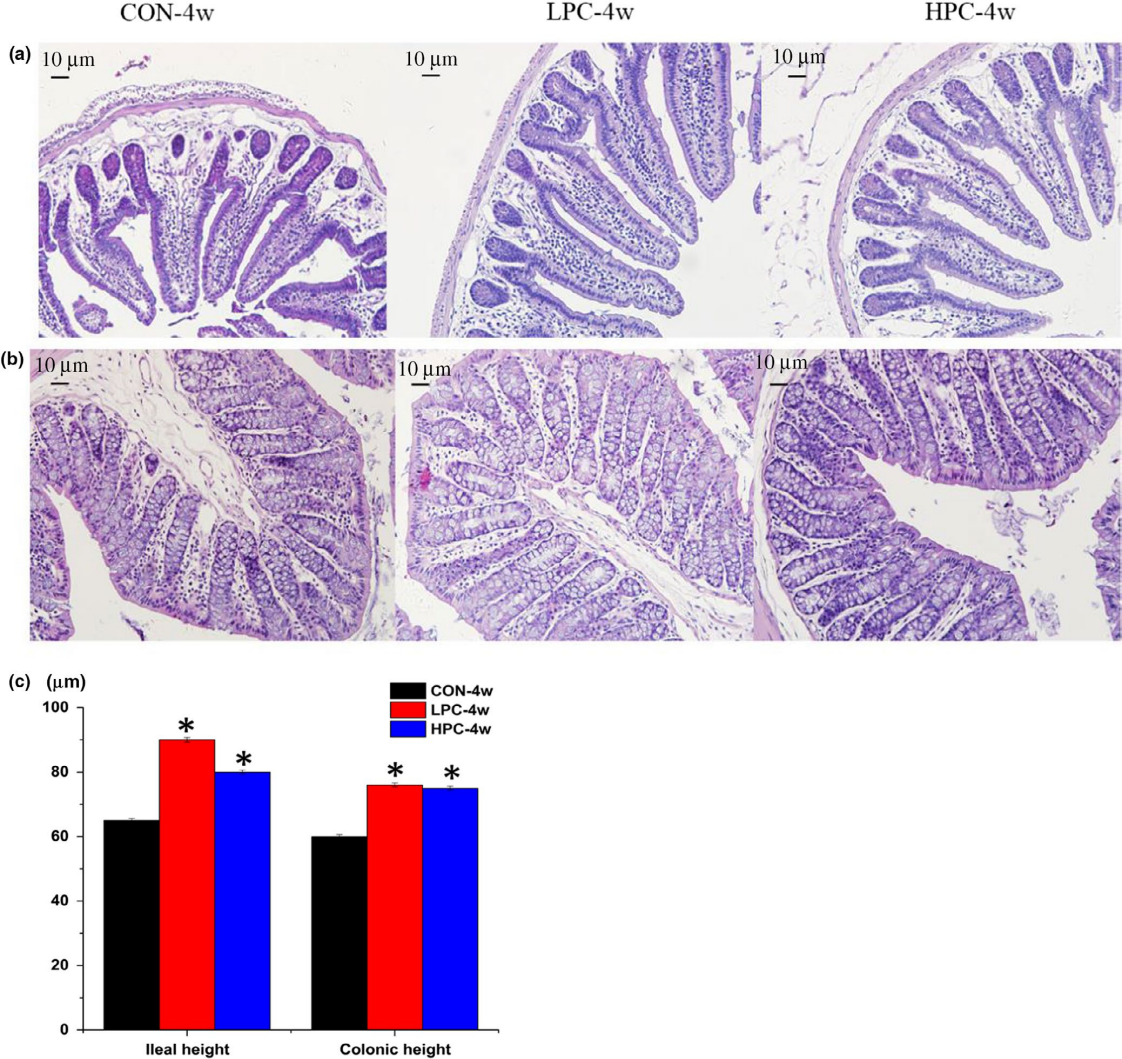
Effects of a PC‐supplemented diet on the intestinal mucosal morphology. (a) Ileal morphology observed with hematoxylin–eosin (HE) staining in the control group and different dietary groups (stained sections were photographed at 200× magnification). (b) Colonic morphology observed with HE in the control group and different dietary groups (stained sections were photographed at 200× magnifications). (c) Villus height was measured. CON‐4w, blank control group, week 4; LPC‐4w, 50 mg/kg PC group, week 4; HPC‐4w, 100 mg/kg PC group, week 4. All data are shown as means ± *SD*s. Data were analyzed by one‐way ANOVA (Tukey's test), *indicates a significant difference compared with the control group (*p* < 0.05)

The effect of dietary PC on the intestinal morphology was assessed by histological examination (Figure 8). The ileal and colonic tissues from the PC groups were more integrated than those from the control group. Compared with the control group, the ileal and colonic tissues in the PC dietary treatment groups showed greater villus height and more intensive goblet cells.

### 3.6 Detection of serum LPS levels

**Figure 9:**
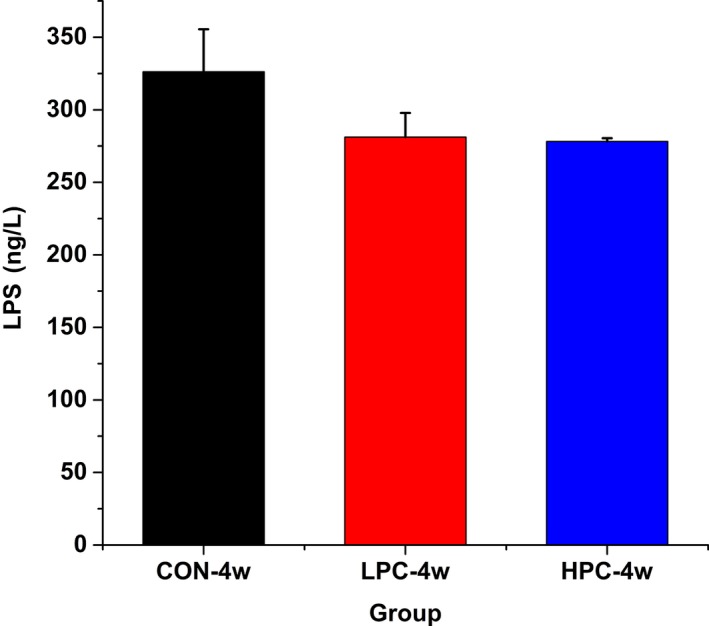
Effect of dietary PC on the serum LPS levels in C57BL/6 mice. CON‐4w, blank control group, week 4; LPC‐4w, 50 mg/kg PC group, week 4; HPC‐4w, 100 mg/kg PC group, week 4 (*n* = 5 per group)

The levels of serum LPS were detected by an ELISA. The average serum LPS level was modestly but not significantly reduced in the PC groups (Figure 9), and these reductions may have correlated with colon permeability.

### 3.7 Correlations of bacterial abundance and mouse phenotypes (LPS level and villus height)

**Figure 10:**
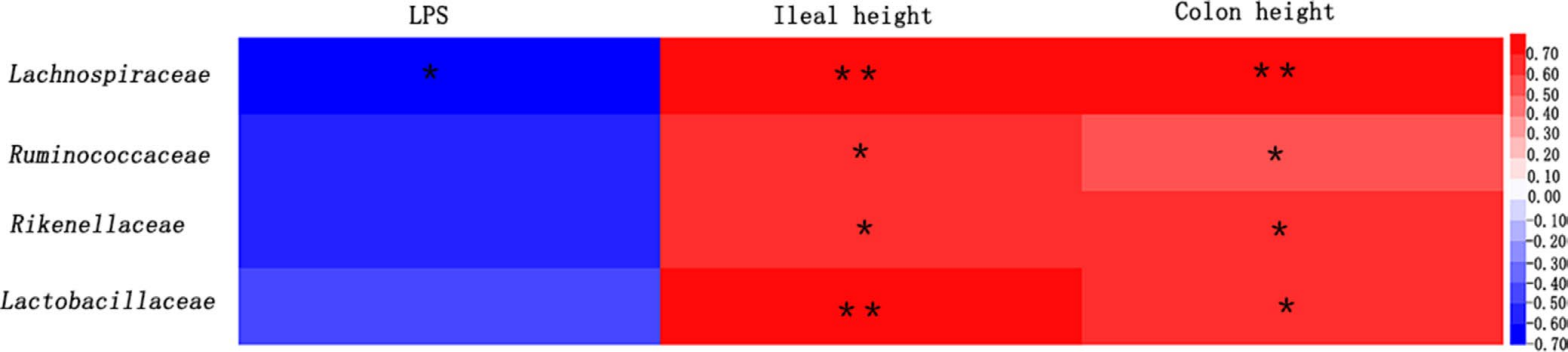
Correlation analysis of bacterial abundance and mouse phenotypes (LPS level and villus height). Red represents a positive correlation and blue represents a negative correlation. The intensity of the colors indicates the degree of correlation between the abundant OTUs and the host parameters. *p < 0.05，**p < 0.01

Spearman's correlation coefficients were calculated between the abundance of the four bacterial families (Lachnospiraceae, Ruminococcaceae, Rikenellaceae, and Lactobacillaceae) and the mouse phenotypes (LPS level and villus height) (Figure 10). LPS was negatively associated with the four bacterial families, whereas the ileal and colonic villus height were positively associated with the four bacterial families.

## 4 DISCUSSION

Phycocyanin, a pharmaceutically active compound, has attracted increasing attention in recent years. Many studies have shown that PC has many health benefits, including antioxidant, immunomodulatory, and anti‐inflammatory activities (Wu et al., 2016). However, its influence on the intestinal microbiota is still unclear. In this study, PC was administered to mice to study its effects on the intestinal microbiota and the gut barrier.

16S rRNA sequences were used to study the effects of PC on the intestinal microbiota. Bacterial abundance and diversity tended to be higher in the PC‐treated groups than in the control group after PC interventions for 4 weeks. Intestinal villi are finger‐like projections located on the intestinal epithelium and constitute the intrinsic layer that bulges toward the lumen of the intestine, from which they absorb nutrients. Phycocyanin treatment increased the length of the intestinal villi. An increase in intestinal villi is conducive to the absorption and utilization of food and increases the amount of bioavailable substances reaching the lower digestive tract. This may explain the increased abundance and diversity of the intestinal microbiota communities in the PC‐treated mice.

The most abundant phyla in the gut were Bacteroidetes and Firmicutes, which accounted for more than 90% of the total bacteria, consistent with previous reports (Zhai, Zhu, Qin, & Li, 2018). Changes in the ratio of Bacteroidetes to Firmicutes significantly affect the host's health (Pyo, Pajarillo, Kyoung, Heebal, & Dae‐Kyung, 2016). In this study, the ratio of Bacteroidetes to Firmicutes decreased significantly after PC interventions for 4 weeks and was lower in the low‐dose PC group than in the high‐dose PC group. Research has shown that the ratio of Bacteroidetes to Firmicutes is lower in obese individuals than in non‐obese individuals (Ley, Turnbaugh, Klein, & Gordon, 2006). This suggests that PC may lead to weight gain. However, we observed no weight differences between the PC groups and the control group, although the 4‐week experimental period may have been too short to observe such differences.

The relative abundance of bacteria in the phylum Deferribacteres increased significantly after PC treatment for 4 weeks (*p* < 0.05). Members of Deferribacteres gain energy through obligate or facultative anaerobic metabolism, and all use iron, manganese, or nitrate for anaerobic respiration. They can also produce energy for their host by fermentation (Huber, Foesel, Pascual, & Overmann, 2017). Bacteria belonging to the Deferribacteraceae family also increased more significantly in the low‐dose PC group than in the high‐dose PC group.

Phycocyanin intervention significantly increased the abundance of the family Lactobacillaceae (in the low‐dose PC group), which had a bifidogenic effect. The family Lactobacillaceae contains well‐known probiotic bacteria, which benefit the host's health. The bifidogenic effect correlated inversely with the PC dose. However, the effects of PC were far more complex than merely a bifidogenic effect, and other microbes in the gut were also affected by PC.

Phycocyanin treatment caused a dramatic increase in the abundance of the families Lachnospiraceae and Ruminococcaceae, and the low‐dose PC group showed more significant effects. Each of these bacterial taxa ferments carbohydrates and produces butyric acid (Zackular, Rogers, & Schloss, 2014) and they play important roles in maintaining gut health (Louis & Flint, 2010). Butyrate is produced by intestinal microbial fermentation (Hamer et al., 2008), and can provide energy directly to the gut epithelium, improving intestinal digestion, the absorption of nutrients, and intestinal immunity (Shangari et al., 2007; Trivedi & Jena, 2013; Vital, Karch, & Pieper, 2017). Members of the family Lachnospiraceae in the genus *Clostridium* are reported to protect their host against colon cancer by producing butyric acid (Meehan & Beiko, 2014). The family Ruminococcaceae, which is associated with butyrate production, is one of the most abundant families in the order Clostridiales, and reportedly maintains intestinal health (Biddle, Stewart, Blanchard, & Leschine, 2013). Therefore, PC may stimulate butyrate production and thus potentially improve gut health, and low‐dose PC had a greater effect than high‐dose PC.

A significant increase in bacteria of the family Rikenellaceae was observed in the PC‐treated groups. Members of the family Rikenellaceae are hydrogen‐producing bacteria that selectively neutralize cytotoxic reactive oxygen species (ROS) and protect cells from oxidative stress (Chen, Zuo, Hai, & Sun, 2011). It has been reported that endogenous hydrogen reduces oxidative stress and ameliorates the symptoms of inflammatory bowel disease (Si, Cheng, Wyckoff, & Qiang, 2016), which would benefit the host's health. Low‐dose PC had a greater effect on the level of Rikenellaceae than the higher dose.

At the genus level, PC intervention significantly increased the abundance of *Alistipes.* *Alistipes* produces succinic acid as the main metabolic end product of glucose fermentation and uses iso‐C15:0 as its main long‐chain fatty acid (Song et al., 2006).

To explain the effects of PC on variations in the intestinal microbiota, we conducted PCoA. After PC intervention, the intestinal microbiota differed significantly between the PC groups and the control group in terms of their structure and diversity.

Our results indicate that, overall, the low dose of PC (50 mg/kg) showed better effects on the regulation of the intestinal microbiota than the high dose (100 mg/kg), but no statistically significant differences were observed. This lack of difference might be because the concentrations (different doses) used in this experiment were both within the optimum range, given the experimental time frame.

The gastrointestinal tract is an important organ located between the external and internal environments. Intestinal epithelial cells are directly affected by their environment, including the host diet, poisons, and microbial metabolites (Lallès, 2017). The intestinal epithelium has a surface area of approximately 100 m^2^, lined with a monolayer of columnar intestinal epithelial cells, which form a thick physical barrier to the entry of food and bacterial substances. This is the first line of defense in the host gut (Maloy & Powrie, 2011). Reduced intestinal permeability can increase intestinal barrier function and prevent the transfer of toxic substances and harmful bacteria into the bloodstream. Phycocyanin treatment increased the numbers of intestinal columnar epithelial cells and goblet cells, and increased the intestinal barrier function. The presence of a thickened colon in the PC‐treated animals was also at least partly supported by the downward trend in the average serum LPS levels, although the differences were not significant, which might be attributable to differences between individuals.

Spearman's correlation coefficients were calculated to determine whether bacterial abundances correlated with mouse phenotypes (LPS level and villus height). The abundances of the four bacterial families correlated negatively with the increase of LPS, and this correlation was significant for Lachnospiraceae. The abundances of the four bacterial families also correlated positively with the ileal and colonic villus height.

## 5 CONCLUSIONS

In this study, we evaluated the effects of PC treatment on the intestinal microbiota and gut permeability in mice. The administration of PC increased the richness and diversity of the intestinal microbiota, perhaps because PC promoted the growth of the intestinal villi, enhancing the digestion and absorption of food, and thereby increasing the nutrients available to the intestinal microbiota. Phycocyanin intervention significantly increased the abundance of the family Lactobacillaceae, which exerts a bifidogenic effect. The family Lactobacillaceae contains well‐known probiotic bacteria, which benefit the host's health. Dietary PC also increased the carbohydrate decomposition and the numbers of short‐chain fatty acid‐producing bacteria. Therefore, PC may improve gut health by stimulating the production of short‐chain fatty acids. The family Rikenellaceae, which contains hydrogen‐producing bacteria that can selectively neutralize cytotoxic ROS and protect cells from oxidative stress, increased after the PC interventions. Dietary PC increased the colonic epithelial barrier function and prevented endotoxins entering the systemic circulation. These modulatory effects were related to the PC dose, with the low‐dose PC group showing better effects than the high‐dose PC group. Further studies are required to determine the roles and underlying mechanisms of PC in modulating other intestinal microbiota. However, our data provide a basis for the future modulation of intestinal microbes by treatment with PC.

## CONFLICT OF INTERESTS

The authors confirm that they have no conflicts of interest.

## AUTHORS CONTRIBUTION

Yuanyuan Xie, Wenjun Li, Song Qin, and Zhenning Du conceived and designed the experiments, and contributed to the writing of the manuscript. Yuanyuan Xie, Limeng Zhu, and Shixiang Zhai conducted the experiments.

## ETHICS STATEMENT

The study design was approved by the Animal Care and Maintenance Committee of Shandong International Biotechnology Park (SCXK 20140007), and adheres to the guidelines of the Institute of Health/Institutional Animal Care and Use Committee.

## Data Availability

The raw data were uploaded to the National Center for Biotechnology Information SRA database under the accession number PRJEB30114.
